# Application of a Novel Tool for Diagnosing Bile Acid Diarrhoea

**DOI:** 10.3390/s130911899

**Published:** 2013-09-06

**Authors:** James A. Covington, Eric W. Westenbrink, Nathalie Ouaret, Ruth Harbord, Catherine Bailey, Nicola O'Connell, James Cullis, Nigel Williams, Chuka U. Nwokolo, Karna D. Bardhan, Ramesh P. Arasaradnam

**Affiliations:** 1 School of Engineering, University of Warwick, Coventry CV4 7AL, UK; E-Mails: E.W.Westenbrink@warwick.ac.uk (E.W.W.); N.Ouaret@warwick.ac.uk (N.O.); 2 MOAC Doctoral Training Centre, University of Warwick, Coventry CV4 7AL, UK; E-Mail: R.Harbord@warwick.ac.uk; 3 Department of Gastroenterology, University Hospital Coventry & Warwickshire, Coventry CV2 2DX, UK; E-Mails: Cathertine.Bailey@uhcw.nhs.uk (C.B.); nicola.o'connell@uhcw.nhs.uk (N.O.); chuka.nwokolo@uhcw.nhs.uk (C.U.N.); R.Arasaradnam@warwick.ac.uk (R.P.A.); 4 Department of Nuclear Medicine, University Hospital Coventry & Warwickshire, Coventry CV2 2DX, UK; E-Mails: james.cullis@uhcw.nhs.uk (J.C.); nigel.r.williams@uhcw.nhs.uk (N.W.); 5 Department of Gastroenterology, Rotherham General Hospital, Rotherham S60 2UD, UK; E-Mail: bardhan.sec@rothgen.nhs.uk; 6 Clinical Sciences Research Institute, University of Warwick, Coventry CV2 2DX, UK

**Keywords:** electronic nose, FAIMS, bile acid diarrhea (BAD), bile acid malabsoprtion (BAM), fermentome

## Abstract

Bile acid diarrhoea (BAD) is a common disease that requires expensive imaging to diagnose. We have tested the efficacy of a new method to identify BAD, based on the detection of differences in volatile organic compounds (VOC) in urine headspace of BAD *vs*. ulcerative colitis and healthy controls. A total of 110 patients were recruited; 23 with BAD, 42 with ulcerative colitis (UC) and 45 controls. Patients with BAD also received standard imaging (Se75HCAT) for confirmation. Urine samples were collected and the headspace analysed using an AlphaMOS Fox 4000 electronic nose in combination with an Owlstone Lonestar Field Asymmetric Ion Mobility Spectrometer (FAIMS). A subset was also tested by gas chromatography, mass spectrometry (GCMS). Linear Discriminant Analysis (LDA) was used to explore both the electronic nose and FAIMS data. LDA showed statistical differences between the groups, with reclassification success rates (using an n-1 approach) at typically 83%. GCMS experiments confirmed these results and showed that patients with BAD had two chemical compounds, 2-propanol and acetamide, that were either not present or were in much reduced quantities in the ulcerative colitis and control samples. We believe that this work may lead to a new tool to diagnose BAD, which is cheaper, quicker and easier that current methods.

## 1. Introduction

Chronic diarrhoea is a significant clinical problem estimated to affect up to 5% of the population [1]. There are many causes for chronic diarrhea, but bile acid diarrhoea (BAD) is one of the commonest [2]. Bile acids are essential for the emulsification and subsequent digestion of fat. More than 98% of bile acids produced are reabsorbed back in to circulation, with less than 2% lost in faeces [3]. If this process of reabsorption is perturbed either through disease, surgical removal of a length of bowel or through defects in certain regulatory proteins, then, the excess bile that is not reabsorbed spills over into the colon resulting in symptoms of diarrhea [4]. The excess bile acids can be ‘mopped up’ or sequestered effectively using medication known as bile acid sequestrants. Although common, BAD is under diagnosed partly as it requires nuclear medicine imaging and ingestion of a radioactive capsule (selenium 75 tagged to homochlorotauric acid—SeHCAT). The SeHCAT retention test involves a synthetically tagged isotope, which is swallowed and diluted within the bile acid circulation and thus able to track its movements. This is the accepted gold standard to diagnose BAD, but the cost is prohibitive—approximately £210 ($320) per patient and thus in some areas patients have been denied the opportunity to have this diagnosis made.

Host gut bacteria are essential in the cleavage of bile acids but its diversity has been difficult to study as less than 50% of organisms can be successfully cultured. Modern genomic techniques can circumvent this problem but are expensive, laborious and not practical for daily clinical use. The study of the resultant products of fermentation—‘the fermentome’ can be measured for various diseases using urine and faeces [5–9]. By examining the signature of some volatiles, we have been able to distinguish those with diarrhoea due to inflammatory bowel disease (a chronic relapsing condition) [10]. The quest continues to find simple, reliable non-invasive markers to identify those with bile acid diarrhoea.

To address this important clinical problem, we propose the utilisation of real-time instruments, which are able to detect VOCs and other gases that emanate from biological waste material. We have specifically tested an electronic nose (an instrument that mimics the biological olfactory system) [11] and also a newer technology—Field Asymmetric Ion Mobility Spectrometer (FAIMS). The FAIMS is an instrument that separates ionized molecules based on their different mobilities in a high electric field [9]. These results are compared to Gas Chromatography, Mass Spectrometry (GCMS) to identify any key chemical differences within the samples. The aim of this study was to test the potential usefulness of these techniques to differentiate between those with bile acid diarrhoea, ulcerative colitis (a disease with similar symptoms but different causality) and controls using only urine samples.

## 2. Experimental Section

All urine samples were analysed using an AlphaMOS Fox 4000 Electronic nose and an Owlstone Lonestar FAIMS and a subset analysed by GCMS. Urine was collected in a standard universal specimen container and immediately stored at −80 °C after collection for subsequent batch analysis. Before testing, the samples were left to thaw overnight in a lab fridge at 4 °C and then aliquoted into appropriate sample bottles (described below). The samples were then used for analysis using the electronic nose and FAIMS experimental methods. FAIMS measurements were undertaken over a 6 month period (tested in three batches) with a sub-group used for a single electronic nose test (due to instrument availability).

### 2.1. Subjects

A total of 110 patients were recruited for this study and consisted of adults aged 28 to 81 years. Patients were recruited from general Gastroenterology and Inflammatory bowel disease clinics at University Hospital Coventry & Warwickshire, UK and Rotherham General Hospital, UK. Demographic data and disease activity score index was collected from the patients. The study cohort contained 3 groups: 23 patients with BAD, 42 patients with UC and 45 healthy controls. Bile acid diarrhoea was defined as those with a Se^75^ homochlorotauric acid test of less than 15% retention at day seven (7d SeHCAT retention). A lower retention value suggests inability to reabsorb bile back into circulation thus resulting in bile acid diarrhoea. The UC patients were all in clinical remission (confirmed by clinical scores and inflammatory markers). Those with an uncertain diagnosis or inconclusive radiological or histological confirmation were excluded from the study. The demographics and clinical parameters of the subjects are shown in Table 1. Scientific and ethical approval was obtained from local Research & Development Department and Warwickshire Ethics Committee (reference number: 09/H1211/38). Written informed consent was obtained from all patients who participated in the study. Patients were not required to have a standardized diet. Though this appears appealing to create a stable baseline, its effect is likely to vary as much within individuals as it would between individuals. This is due to the diversity of the gut microbial milieu, which is large. It is estimated that there are ten times more microbes than there are cells in the body, encompassing several major classes and species. Crucially each individual's microbiome is unique. The organisms ferment undigested fibre releasing gases and chemicals both volatile and otherwise. These reflect the interaction of diet and the individual's unique microbes, the person's “fermentation signature”, thus fixing a persons diet would modulate this fermentation signature. Furthermore, adherence to a “regular diet” (without major deviation) is feasible for short periods in volunteer studies, but would be difficult amongst patients, not least because we don't know how long they would have had to be on it before samples are collected.

### 2.2. Electronic Nose

The electronic nose was developed as an instrument to mimic the biological olfactory system and as an alternative to more sophisticated analytical equipment (such as GCMS). These instruments operate at room temperature, use air as the carrier gas and can give a real-time result. They comprise of an array of chemical sensors, where each sensor is broadly tuned to a different gas or vapour. When a complex sample is presented to the chemical sensor array, as each sensor is different, the response of each sensor to the sample is unique. The response of all the sensors can be brought together to create a smell ‘fingerprint’ of that sample. When a similar sample is presented again to the instrument, it will produce the same sensor response fingerprint and thus we are able to identify that sample. This identification process is normally achieved using some form pattern recognition technique. Consequently, we are able to present many different types of sample to the instrument, allow it to learn these smell fingerprints, and then characterize/identify new samples. In this study a commercial electronic nose (Fox 4000, AlphaMOS, Toulouse, France) was used to analyse the chemical signature of the urine samples. This instrument comprises of an array of 18 metal oxide gas sensors, whose resistance is modulated in the presence of a target gas/vapour.

Experimentally, 5 mL of each urine sample were aliquoted into 10 mL bottles. The Fox 4000 electronic nose is fitted with a HS100 autosampler, which allows up to 64 samples to be run in one batch. The autosampler first moves each bottle into a preparation chamber, which heats the sample for 10 min to 60 °C and agitates the bottle. After 10 min a syringe takes 1 mL of headspace from the sample bottle and directly injects it into the electronic nose. The change in resistance of the sensors was measured from the injection time for 180 s at a sample rate of 1 Hz. The instrument was flushed with clean, dry air (flow rate of 500 mL/min) for 10 min after each exposure to ensure that the sensors had fully recovered. Each sample was tested three times. Here, a smaller set of samples were tested than with the FAIMS instrument (41 in total, 14 BAD, 20 ulcerative colitis and 7 controls).

### 2.3. FAIMS (Field Asymmetric Ion Mobility Spectrometry)

For FAIMS testing, again a commercial instrument was deployed (Lonestar, Owlstone, Cambridge, UK). Unlike the electronic nose, this system achieves separation of chemical components on the basis of differences in ion mobility within a high electric field. FAIMS is a fairly recent technology that allows gas molecules to be separated and analysed at atmospheric pressure and room temperature. Here a test sample is first ionised with a radiation source (Ni-63 in our case), resulting in a group of ions of various sizes and types. These are introduced between two conductive plates and an asynchronous waveform is used, where a high positive voltage is applied for a short time and a low negative voltage is applied for a longer time, but their magnitudes equal in terms of voltage × time. The ionized molecules are subjected to these high electric fields and depending on their physical properties, move towards or away from one of the high voltage plate (or not affected at all) depending upon their mobilities. If ions touch either of the plates, their charge is lost and not detected as they exit the plates. Therefore, a compensation voltage is added that counteracts a specific level of mobility allowing those molecules to exit the plates with their charge and be detected. By scanning through a range of compensation voltages, a range molecules will different mobilities can be measured [9].

For FAIMS, 7 mL of urine was aliquoted into a standard 30 mL Sterilin bottle (Newport, UK). The plastic lids were modified with the addition of push-fit fittings (for 3 mm PTFE tubing), which allowed the bottle to be connected to the FAIMS instrument. The sterilin bottles were heated to 60 °C ± 0.1 for 30 min before each experiment. The FAIMS instrument was set up in a pressurised configuration with a flow rate of 2 L/min. The dispersion field was stepped through 51 equal settings between 0 and 90% (the dispersion field in the ratio of the high electric field to low electric field) and for each dispersion field the compensation voltage stepped was between +6 V and −6 V in 512 steps. Both positive and negative scans were used (electric fields applied first with a high positive potential and then with a high negative potential), where each scan produced 26,112 data points.

### 2.4. Statistical Methods

Exploratory data analysis for the electronic nose was performed using Principal Component Analysis (PCA) and both the electronic nose and FAIMS using Linear Discriminant Analysis (LDA). These exploratory techniques are extensively used for these types of experiment. Their purpose is to allow the simple interpretation of complex data to determine if differences in groups of samples can be seen. For the electronic nose analysis, the raw data was extracted using Alphasoft (AlphaMOS v12.36) and analysed in Multisens Analyzer (JLM Innovations, Tübingen, Germany).

FAIMS data was processed in a custom LabVIEW program (Ver. 2012, National Instruments, Austin, TX, USA). For analysis, both the positive and negative ion matrices for each scan were concatenated and joined to make a single 52,224 element array. These were then wavelet transformed using a Daubechies D4 wavelet. Variables in the resulting array, suitable for discrimination, were then identified. For each variable, the class scatter (Σσ_i_)^2^ and the between class scatter: (σ_μ_)^2^/(Σσ_i_)^2^, were calculated and then thresholds set to identify variables for analysis. (σ_i_: the standard deviation of the dimension in question within the class i, and σ_μ_ was the standard deviation of the means of the dimension under test between classes). These were then used as the input to a LDA algorithm.

This approach gave a two dimensional input parameter space (within class scatter and between class scatter) to control the separation algorithm. This space was explored by incrementing through threshold values of these two parameters and selecting variables that were below this threshold. For each threshold increment, one sample was removed from the dataset and re-classification was attempted based on the analysis of the remaining samples. Classification employed a K-Nearest-Neighbour (KNN) routine. This exploration identified groups of common variables in the parameter space where re-classification exceeded that which would be expected from random re-classification (three standard deviations from the mean). Variables set in this robust region were used for further analysis.

### 2.5. Gas Chromatography/Mass Spectrometry

A subset of the samples (10 from each group; BAD, ulcerative colitis and controls) were analyzed by GCMS to evaluate if there were any key chemical markers for bile acid diarrhoea. Here analysis was undertaken using a Bruker Scion SQ GCMS system, fitted with a Restek Rxi-624Sil MS fused silica GC column (length 20 m, 0.18 mm internal diameter, 1.0 μm wall thickness) and a Combipal Autosampler (CTC, Zwingen, Switzerland). Due to the expected small concentrations of chemical components within the sample, the autosampler was enhanced with an ITEX2 pre-concentrator (CTC). Five mL of urine sample was aliquoted into 10 mL glass vials and the lid crimped. Samples were heated to 60 °C and the headspace pulled through the ITEX a total of 15 times per sample over a time period of 3 min. After pre-concentration the sample was desorbed into the GCMS by heating the ITEX up to 250 °C and injecting into the fused silica column. The injector was kept at a constant temperature of 250 °C, sending samples in the column at a split ratio of 1:20 to maintain peak sharpness at the end detector. The GC then underwent a temperature program in order to separate the samples' constituent VOCs in terms of boiling point and molecular weight, holding at 50 °C for 1 min before increasing at a constant rate of 20 °C/s up to a maximum of 280 °C. The separated species were detected by the chromatograph, then fragmented and analysed by the mass spectrometer.

## 3. Results and Discussion

### 3.1. Electronic Nose, FAIMS and GCMS Results

**Figure 1.**
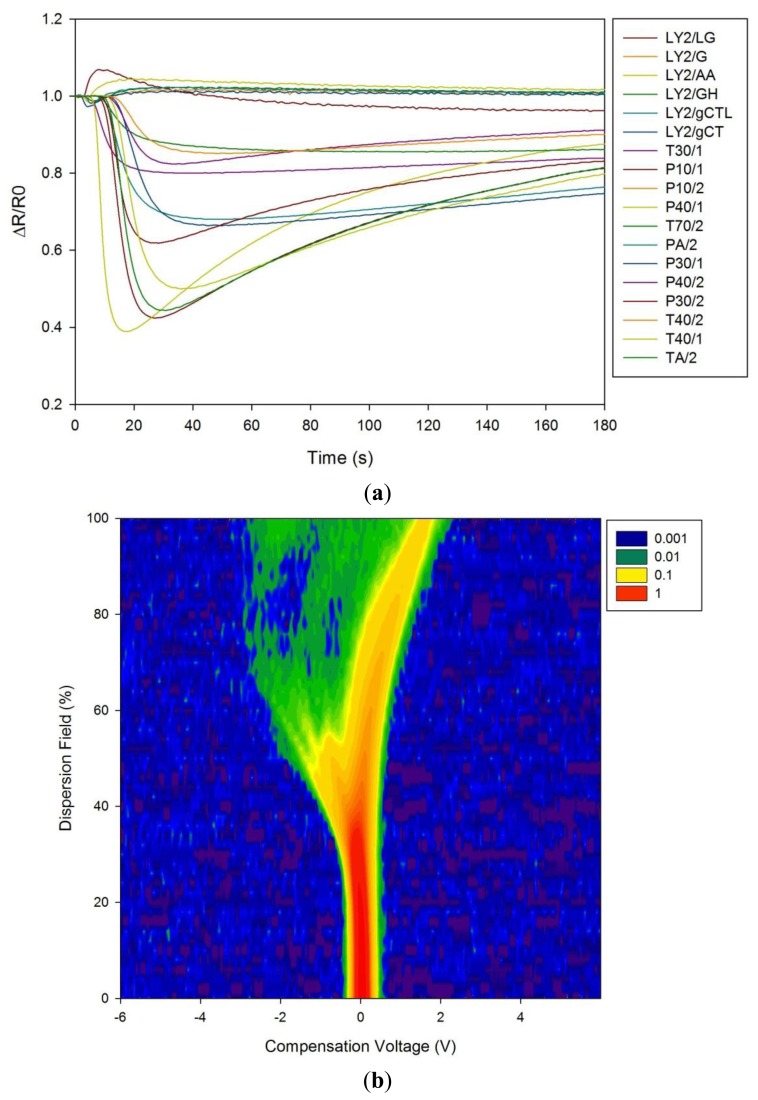
(**a**) Raw electronic nose results showing the sensor responses to a BAD patient urine sample. (**b**) Raw data from the FAIMS instrument to a BAD patient urine sample. Intensity is in arbitrary units of ion count.

**Figure 2.**
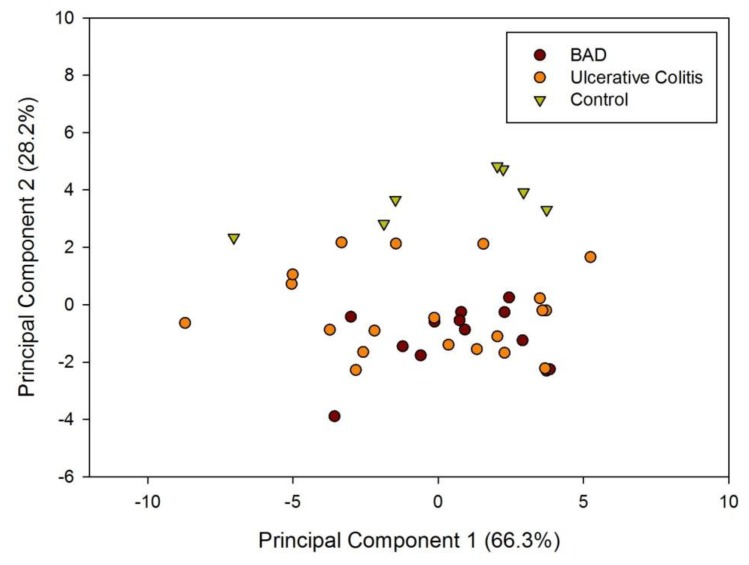
Principal Component Analysis of AlphaMOS Fox 4000 results.

**Figure 3.**
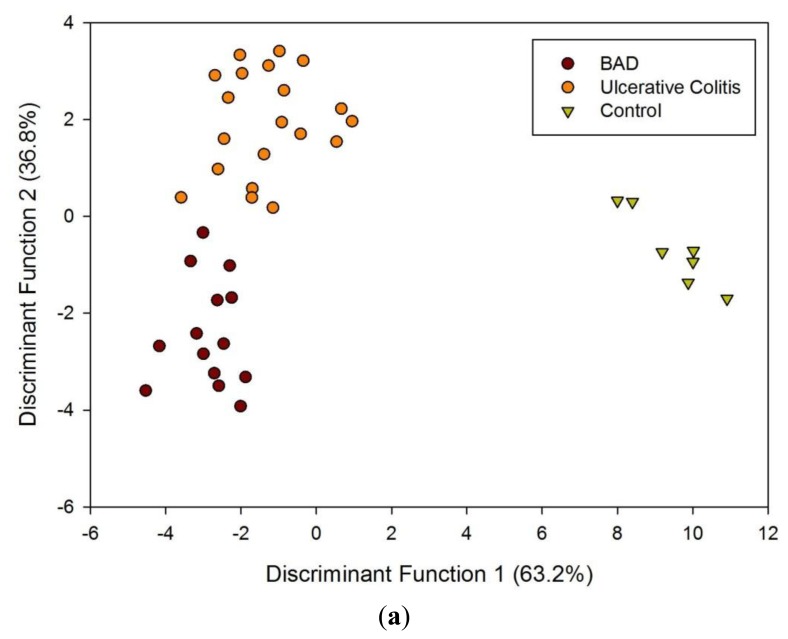

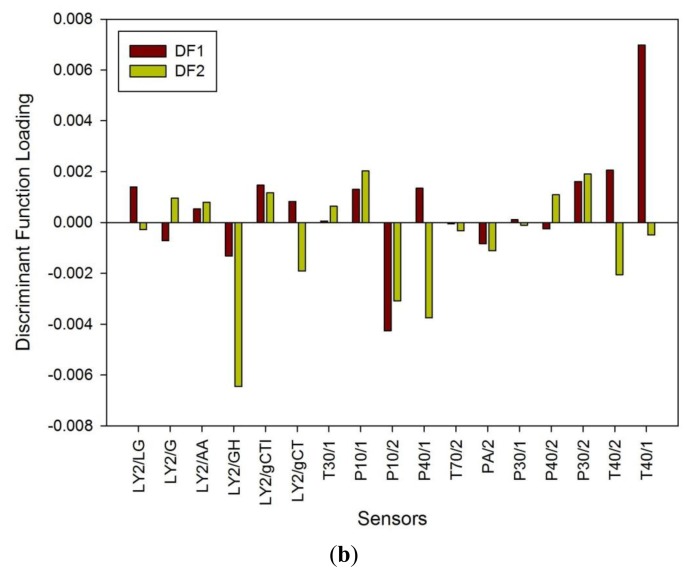
(**a**) Linear Discriminant Analysis of AlphaMOS Fox 4000 results. (**b**) Associated loadings plot for LDA (DF is discriminant function).

Figure 1a shows a typical response of the electronic nose sensor array to a urine sample and Figure 1b shows a typical FAIMS ‘plume’ for a positive ion scan. The maximum change in resistance was used as the feature for data processing for the Fox 4000 and change in total ion current for the Lonestar. Initial electronic nose analysis was undertaken using PCA (PCA being a non-classified technique). Results from PCA showed no clear trend, as shown in Figure 2. Here only the control group shows some slight separation. However, when the samples are re-analysed using LDA (a pre-classified technique), shown in Figure 3a, the groups are clearly separated. Figure 3b displays the associated loadings for this plot, showing that the analysis employs a spectrum of different sensors. Test sets were removed and reclassified (using an n-1 algorithm and a K-nearest neighbour reclassification routine based on LDA weights). Accuracy of reclassification was 85% for the three groups (taken together due to the small sample size).

**Figure 4.**
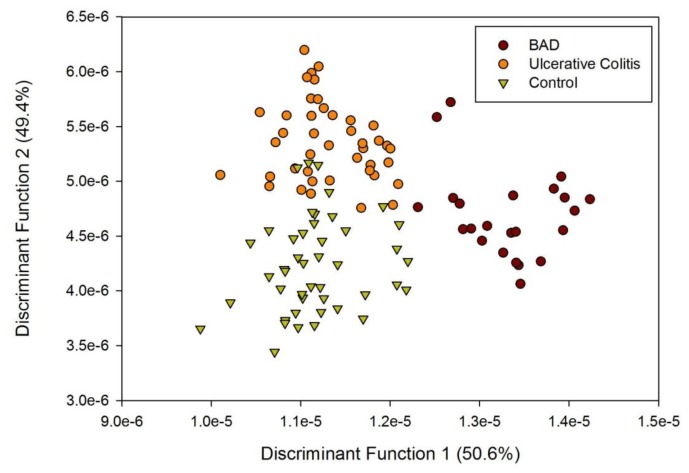
Linear Discriminant Analysis of FAIMS data.

The results of LDA on the FAIMS data for patients with BAD, ulcerative colitis and controls are shown in Figure 4. Using a pre-classified technique (LDA) and employing the cluster analysis methods described earlier, we are able to see clear patterns within the data. Accuracy of reclassification of the elements of the test sets varied between the different sample groups, with controls being 82%, ulcerative colitis being 79% and BAM 83%. However, re-classifying based on BAM and non-BAM results is a reclassification above 90%. This indicates a significant difference in the spectra associated with disease/control groups, but the optimum set of variables are yet to be identified.

**Figure 5.**
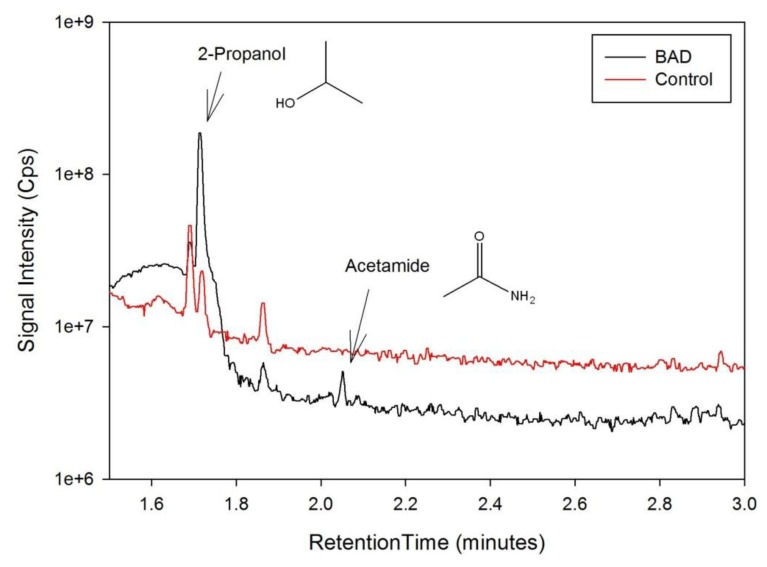
Section of a BAD sample GC Chromatogram showing unique peaks.

**Figure 6.**
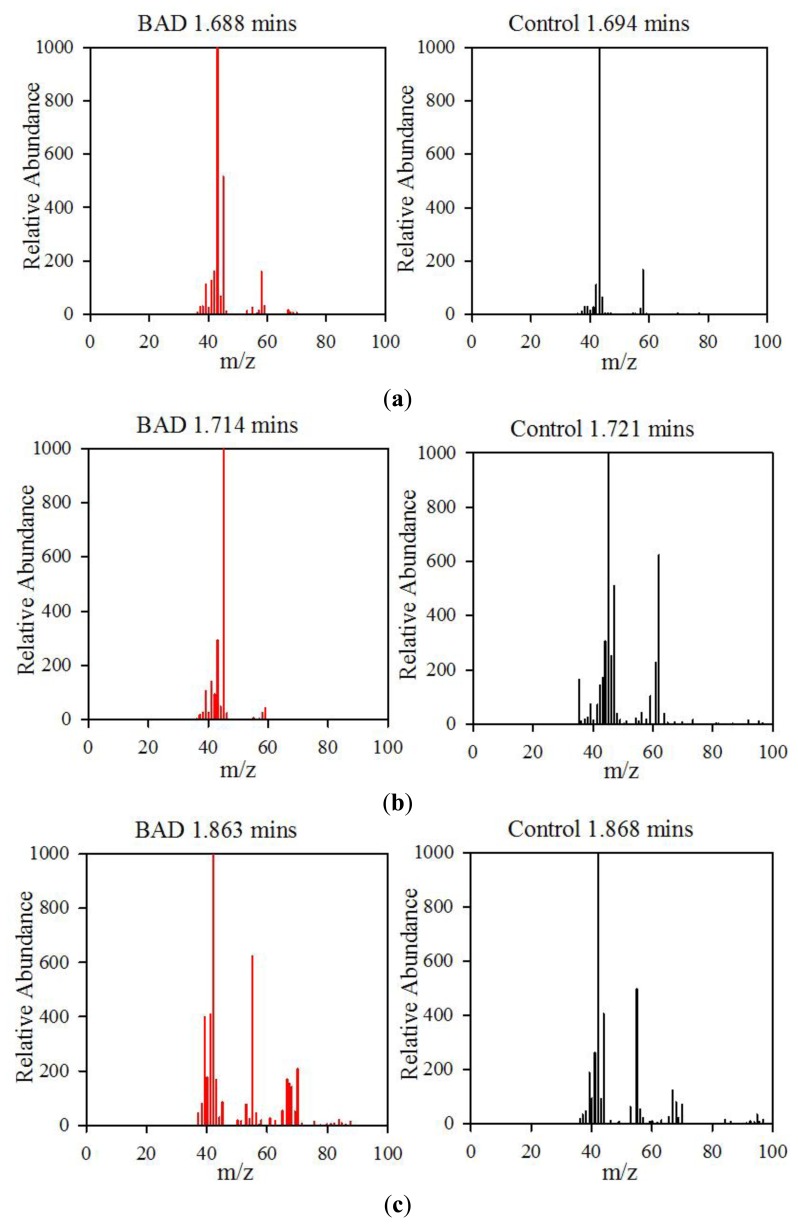

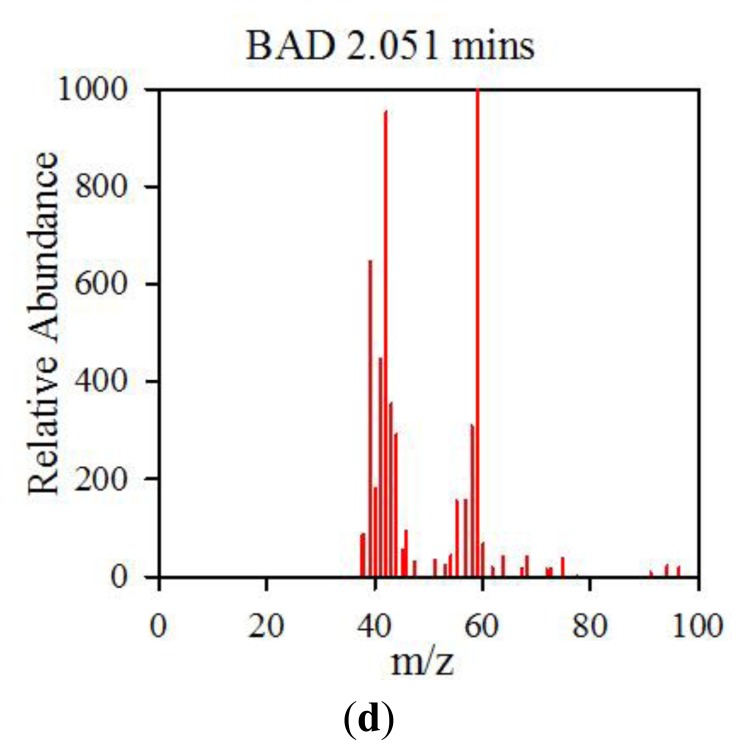
Comparison of BAD and Control Mass Spectra at similar Retention Times. (**a**) These spectra show similar mass ion ratios at 43 and 58, indicating the groups of Acetone. (**b**) These have similar mass ion peaks at and around 45, potentially indicating an alcohol as a common root. However, BAD yields much higher concentration, and there are extra mass ion peaks present in controls. (**c**) These have similar mass ion ratios for most significant peaks (42, 55, 41, *etc*.), indicating a common root. (**d**) This shows a unique set of mass ion ratios not found in controls, with a peak at 59 indicating an amide.

The data from the GCMS was analysed by comparing the retention times (from the gas chromatography) and molecular mass (from the mass spectrometry) observed by the instrument with those from a known NIST library of chemical compounds (NIST 2012). While individual sample variation was high, some overall trends in concentration ratios of the constituent gases were observed which separated the BAD samples from the ulcerative colitis and controls. Most notably, two peaks were found within the BAD samples that were not noticeably present in any other sample groups. These were observed at 1.71 min and 2.05 min in BAD sample chromatograms (as shown in Figure 5), and are consistent with NIST library entries for 2-propanol and acetamide, respectively. There are two pairs of mass ion spectra associated with the GC peaks of both samples in this region (shown in Table 2) that have very similar mass ratios and retention times, and therefore are likely caused by the same compounds. The peaks present at 1.71 min have similar mass ion peaks at 45, which potentially indicate they have an alcohol group in common. However, they have significantly different mass ratios, potentially caused by chemical groups that are unrelated, as shown by the differing NIST identifications. The group in the BAD sample is also at a much higher concentration due to the size of peak produced and is very clearly identified as Isopropyl Alcohol. Finally, the GC peak at a retention time of 2.05 min is not seen at all on chromatograms from samples that are not from BAM patients. These mass spectra are illustrated in full in Figure 6. It is possible that these compounds relate to products of fermentation rather than effect of drugs but requires further investigation.

### 3.2. Discussion

Our study, for the first time, demonstrates the utility of novel non-invasive technologies for use in a common clinical condition of chronic diarrhoea. Specifically, the capability of the methods (described here) for discriminating between urine samples from patients with the less common form of diarrhoea associated with inflammation (ulcerative colitis) and the more common form due to bile acid diarrhoea. This distinction is important to make for two reasons: (1) the clinical management is very different for both these conditions and (2) therapeutic efficacy that is offered is effective especially as newer therapies (bile acid resins) are becoming available suggesting a greater number of people are likely to gain benefit if the condition can be diagnosed.

The VOC/gas signature profiles are different between those with bile acid diarrhoea, ulcerative colitis (type of inflammatory bowel disease) and healthy individuals being detected using two distinct types of technologies namely electronic nose and ion mobility spectroscopy—FAIMS.

The uniqueness of our study is the specific use of non-invasive tests which to our knowledge is the first to demonstrate the use of VOCs detected by electronic nose and FAIMS technology in urine of patients with bile acid diarrhoea. We have previously showed its utility in detecting those with inflammatory bowel disease as well as those with cancer subjected to pelvic radiotherapy using the electronic nose and FAIMS [8,12].

The changes in gas profile in patients with BAD compared with Ulcerative Colitis and healthy controls confirms our *a priori* hypothesis that gut dysbiosis in those with BAD results in a chemical fingerprint that can be detected (altered fermentation profile); much the same as we have demonstrated in those with inflammatory bowel disease where gut dysbiosis has been shown [13]. VOCs and other vapours are produced as a result of colonic fermentation following a complex interaction between the colonocyte, human faecal flora, and mucosal integrity and invading pathogens [5]. They are emitted from bodily fluids and as a result, vapours emitted from urine, faeces and breath may include biomarkers of use in the assessment of gastrointestinal disease. Therefore changes in the gas profile are reflective of the variation of gut microbiota and shed light onto their causative role in pathological states [10]. It is plausible that the biodiversity of gut microflora, which cleave bile acids, may be altered in those with bile acid diarrhoea resulting in changes in the gas profiles of these patients—an indirect representation of the complex pathological process involved in the disease state.

This pilot data shows that we can distinguish diarrhoea due to excess bile acids compared to those with inflammatory conditions. Confirmation was noted by GCMS where 2-propanol and acetamide were found to be prominent only in patients with BAD suggesting that these compounds could possibly be used to distinguish BAD from other disease groups. It is noteworthy that these compounds are not breakdown products of drugs but rather may reflect an altered fermentome profile which is disease specific. The next step will require a specific study design that (a) would involve a larger cohort of ‘pre-diagnosed’ patients with BAD to determine predictive values against the current gold standard technique (*i.e.*, extension of this study) and (b) consequent external validation in newly recruited patients presenting with symptoms that would aid clinicians in a ‘diarrhoea’ diagnostic pathway.

In regard to which technology is the most promising for disease identification, it is hard to state. FAIMS is a more sensitive technology and provides a much higher level of information content. The Fox is simpler to use and the core sensing technology (metal-oxide gas sensors) could offer an easier route to tool development, but sensor drift and sensor to sensor variation may make this challenging. This question can only be answered by analyzing a significantly larger dataset by these technologies (and others) to find the most effective. We hope to undertake this study in the near future.

## 4. Conclusions/Outlook

Current diagnostic methods to diagnose those with BAD require support from nuclear medicine—Se^75^HCAT study, which is expensive and not widely available. The utility of a portable, inexpensive electronic nose that can make a rapid diagnosis in real time will be a paradigm shift in the management of patients with BAD. In this study 110 patients with BAD, ulcerative colitis and healthy controls have been analysed by the electronic nose and FAIMS instruments. Results indicate differences in chemical signatures from these groups, with a re-classification robustness test exceeding 80% in most cases.

## Figures and Tables

**Table 1.** Demographic and Clinical Characteristics of the Study Population.

**Patient Groups**	**Bile Acid Diarrhoea**	**Ulcerative Colitis**	**Healthy Controls**
**Number**	23	42	45
**Age**	49 (14)	56 (15.8)	32(8)
**Sex: M/F**	10/13	10/10	5/9
**BMI**	24 (5)	28 (4.3)	25 (5)
**7 d Se^75^HCAT retention**	7 (1)	n/a	n/a
**5-Aminosalicylate acid (%)**	n/a	33 (85%)	n/a
**Azathioprine (%)**	n/a	7 (17%)	n/a
**Steroids (%)**	n/a	7 (17%)	n/a

Age and Body mass index (BMI) are mean values with standard deviation in brackets. 7d Se^75^HCAT retention are mean retention values at day 7 with standard deviation in brackets. 5-Aminoslicylate acid, azathioprine and steroids are drugs used to treat inflammatory bowel disease (for UC patients), inducing and maintaining clinical remission. Figures are absolute numbers of patients, with percentages of the total number given in brackets.

**Table 2.** Mass Ion Peaks and NIST Identifications of GC Peaks shown in Figure 6.

**Sample**	**GC Retention Time (mins)**	**Mass Ion Peaks (Proportion compared to highest peak)**	**NIST Identifications**
BAD	1.688	**43**(999), **58**(167), **42**(112)	Acetone, dimethyl diazene
Control	1.694	**43**(999), **45**(514), **42**(161), **58**(158), **41**(126)	Acetone, 1-methylethyl hydroperoxide
BAD	1.714	**45**(999), **43**(270), **41**(137), **39**(101)	Isopropyl alcohol
Control	1.721	**45**(999), **62**(624), **47**(508), **44**(307), **46**(252), **61**(227), **43**(172), **35**(165), **42**(141), **59**(103)	Dimethyl disulphide
BAD	1.863	**42**(999), **55**(623), **41**(409), **39**(399), **70**(207), **40**(178), **67**(169), **43**(166), **68**(141)	Cyclopentane, 2-pentene
Control	1.868	**42**(999), **55**(643), **41**(450), **67**(253), **44**(224), **39**(187), **43**(151), **70**(111), **56**(108), **68**(102)	Dihydro-3-methyl-2,5-furandione, cyclopentane
BAD	2.051	**59**(999), **44**(992), **42**(783), **43**(658), **39**(549), **41**(413), **58**(345), **40**(255), **57**(238), **64**(146)	Acetamide
